# Supplementary material for: Discovery of AI-2 Quorum Sensing Inhibitors Targeting the LsrK/HPr Protein–Protein Interaction Site by Molecular Dynamics Simulation, Virtual Screening, and Bioassay Evaluation
Source: Pharmaceuticals (Basel). 2023 May 12;16(5):737. doi: 10.3390/ph16050737 (PMC10223898; doi:10.3390/ph16050737)
Supplement: Supplementary file 1 [file pharmaceuticals-16-00737-s001.zip › pharmaceuticals-2347566-supplementary.pdf]

Table S1. Interactions between LsrK and HPr.

HPr Residues	LsrK Residues	Distance (Å)	Specific Interactions	Hydrogen Bond (HB)	Salt Bridge
Hip 15	Glu 122	2.3	1× HB to Glu 122	1	0
Hip 15	Arg 118	2.6		0	0
Hip 15	Glu 125	3.3		0	0
Thr 16	Leu 126	3		0	0
Thr 16	Glu 122	2.1	1× HB to Glu 122	1	0
Thr 16	Leu 152	3		0	0
Thr 16	Hip 156	2.8		0	0
Arg 17	Glu 125	2.5		0	0
Arg 17	Leu 126	3.5		0	0
Arg 17	Asn 129	3.7		0	0
Ala 20	Leu 126	2.4		0	0
Lys 27	Asp 209	3.4		0	0
Lys 27	Ala 206	4		0	0
Lys 27	Met 210	3.8		0	0
Lys 40	Arg 163	2.6		0	0
Lys 40	Asp 160	2.3	1× HB to Asp 160<br>1× salt bridge to Asp 160	1	1
Ser 41	Arg 163	2.5		0	0
Ala 42	Arg 163	3.5		0	0
Lys 45	Asp 209	3.8		0	0
Ser 46	Met 210	2.5		0	0
Ser 46	Asp 209	2.4	1× HB to Asp 209	1	0
Ser 46	Gly 212	2.6		0	0
Ser 46	Ala 211	3.2		0	0
Leu 47	Met 210	2.4		0	0
Leu 47	Leu 126	2.3		0	0
Leu 47	Leu 152	3.5		0	0
Leu 47	Leu 123	3.7		0	0
Phe 48	Met 210	2.1	1× HB to Met 210	1	0
Phe 48	Leu 152	2.7		0	0
Phe 48	Tyr 162	2.4		0	0
Phe 48	Ala 155	2.4		0	0
Phe 48	Ala 211	2.3		0	0
Phe 48	Leu 151	2.2		0	0
Phe 48	Leu 123	2.2		0	0
Phe 48	Ile 148	2.2		0	0
Phe 48	Leu 126	3.9		0	0
Lys 49	Tyr 162	2.1	1× HB to Tyr 162	1	0
Lys 49	Gly 212	2.2		0	0
Lys 49	Ala 211	3		0	0
Lys 49	Arg 163	2.3	1× HB to Arg 163	1	0
Lys 49	Ala 165	1.9	1× HB to Ala 165	1	0
Lys 49	Met 210	3.7		0	0
Lys 49	Leu 213	3.2		0	0
Lys 49	Ser 166	3.7		0	0
Lys 49	Gln 164	3.9		0	0
Gln 51	Ala 155	3.2		0	0
Gln 51	Hip 156	2.2	1× HB to Hip 156	1	0
Gln 51	Ser 159	3.9		0	0
Gln 51	Leu 152	3.1		0	0
Thr 52	Ala 155	2.2		0	0
Thr 52	Ser 159	2.5		0	0
Thr 52	Arg 163	2.1	2× HBs to Arg 163	2	0
Thr 52	Tyr 162	2.3		0	0
Leu 53	Arg 163	2.4		0	0

Table S2. Information about the 62 compounds obtained by virtual screening. Compounds were tested at 200 μM, and are sorted in the table according to their inhibitory effect. Inhibition rate (%) represents the mean ± SD from three independent experiments (n = 3). The cLogP represents “calculated LogP”.

Compound ID	XP Gscore	Structure	Molecular Weight	cLogP	Inhibition %
4171-0375	-7.308	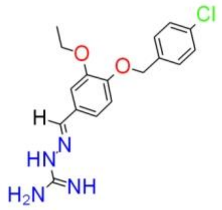	346.82	4.48	98 ± 7
4929-0003	-9.916	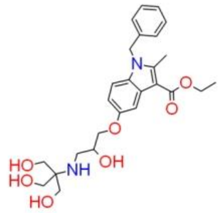	486.57	1.94	78 ± 12
K659-0421	-7.676	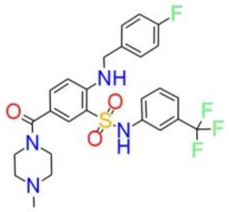	550.57	3.95	75 ± 8
8020-4294	-8.247	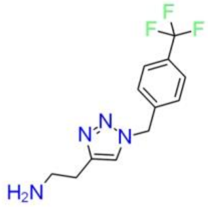	270.26	1.36	65 ± 9
3229-1889	-7.641	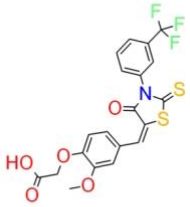	469.45	4.99	63 ± 4
8012-5390	-7.962	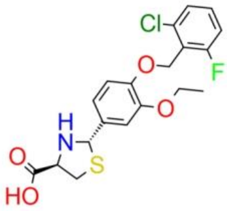	411.87	4.69	60 ± 4

G268-0878	-7.665	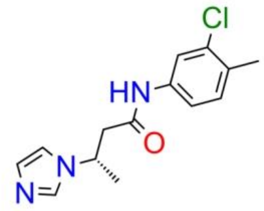	277.75	1.78	58 ± 5
D715-0257	-8.11	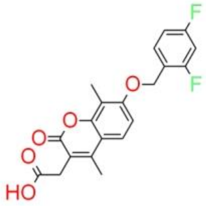	374.34	4.41	57 ± 7
7202-2689	-7.73	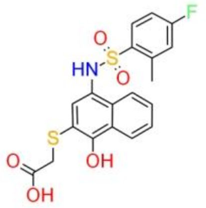	421.46	3.65	49 ± 7
7706-0411	-7.763	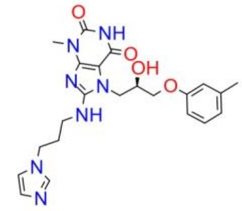	453.50	1.15	48 ± 11
8018-9114	-7.854	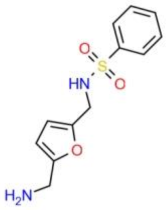	266.32	-0.02	47 ± 10
Y300-1414	-7.387	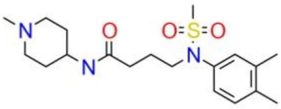	381.21	2.21	47 ± 11
K280-0515	-7.591	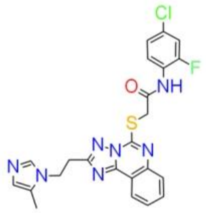	495.96	4.12	46 ± 7
4513-1279	-7.754	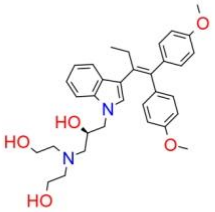	544.69	4.66	45 ± 6

3843-0108	-8.244	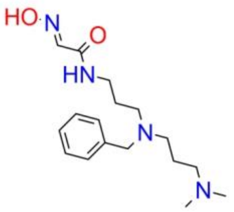	320.22	1.62	42 ± 8
6295-0013	-7.295	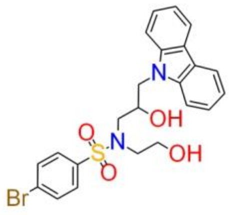	503.41	3.99	42 ± 7
G377-0098	-7.712	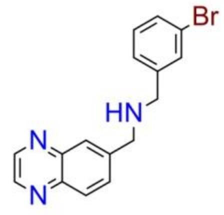	328.21	3.10	40 ± 11
8010-7721	-7.338	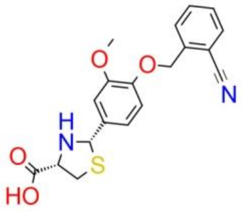	370.42	3.42	37 ± 18
8388-1001	-7.61	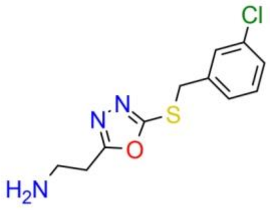	269.75	2.64	35 ± 6
3346-2730	-7.481	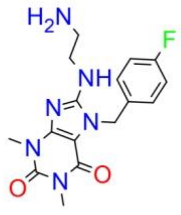	346.37	2.01	35 ± 17
D175-0139	-8.129	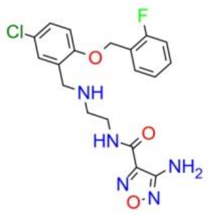	419.84	3.02	33 ± 12
K788-1111	-7.31	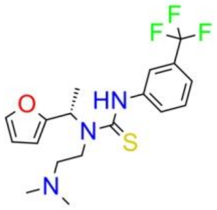	385.45	4.55	32 ± 5

Y020-8649	-7.878	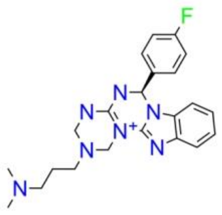	408.23	4.39	30 ± 6
5227-1023	-7.886	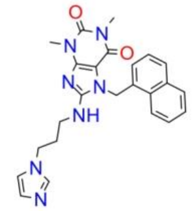	443.51	2.24	30± 14
1501-0340	-7.236	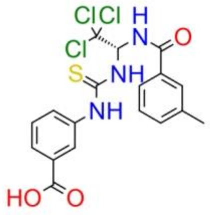	460.75	5.31	26 ± 3
K786-5522	-7.2	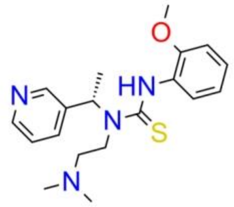	358.50	3.70	25 ± 2
D072-0818	-8.371	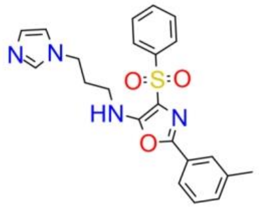	422.50	2.68	24 ± 8
C301-9312	-7.246	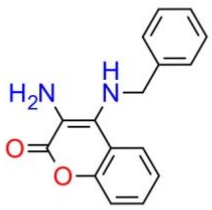	266.30	2.41	24 ± 13
4191-2801	-7.18	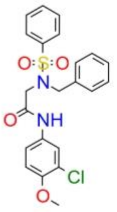	444.93	3.81	24 ± 5
3909-8594	-7.368	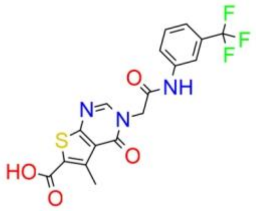	411.36	2.22	22 ± 10

4295-0508	-7.353	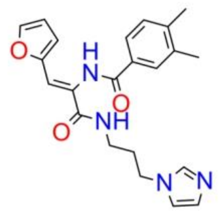	392.46	0.95	18 ± 9
Y041-2219	-7.383	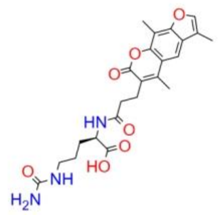	457.48	0.51	18 ± 10
V007-2238	-7.944	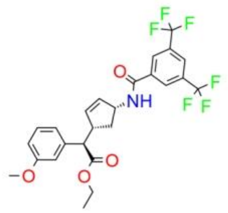	515.45	5.01	17 ± 7
8012-7092	-7.006	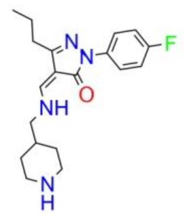	344.43	2.52	13 ± 5
K788-1104	-7.331	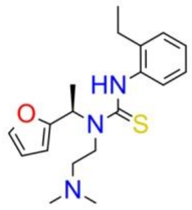	345.51	4.55	11 ± 3
8016-3683	-7.998	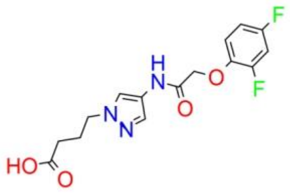	339.30	0.98	10 ± 2
E600-0605	-7.029	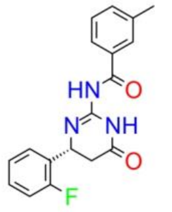	325.34	3.10	10 ± 1
K284-4782	-7.398	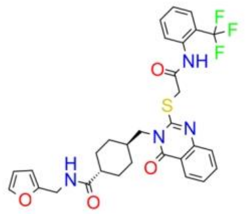	598.64	4.98	10 ± 4

7706-0417	-8.687	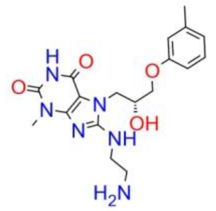	388.43	1.63	9 ± 7
6623-2055	-8.312	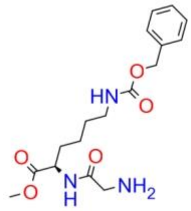	351.40	2.51	8 ± 6
E957-0696	-7.93	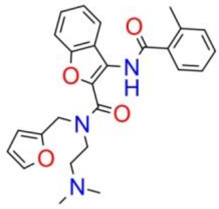	445.52	3.46	7 ± 4
C547-0698	-7.421	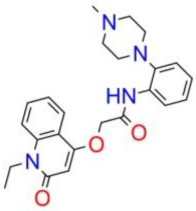	420.51	1.96	5 ± 3
5585-0279	-7.738	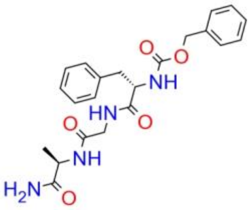	426.47	1.10	4 ± 1
8004-4209	-7.198	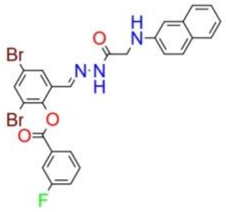	599.25	6.11	4 ± 2
K227-0045	-8.05	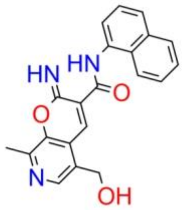	359.13	2.14	0
G935-3933	-8.122	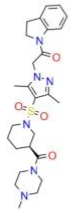	528.25	0.92	0

3254-1114	-7.065	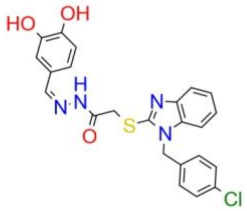	466.94	5.05	0
C312-1678	-7.583	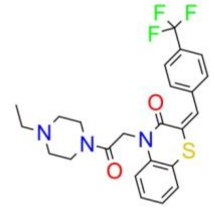	475.53	3.80	0
C328-0147	-7.959	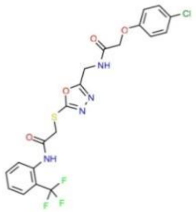	500.88	3.35	0
F594-0352	-7.496	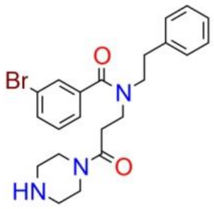	444.37	3.16	0
F919-0042	-8.472	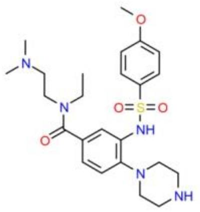	489.64	2.12	0
FF01-5568	-7.03	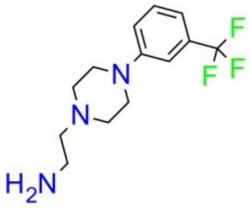	273.30	2.53	0
G718-1201	-7.526	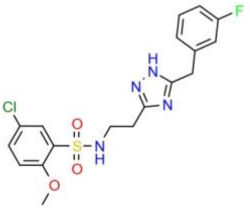	424.88	2.93	0
G765-0111	-7.413	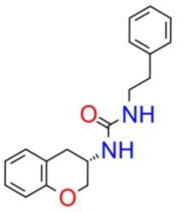	296.37	3.01	0

G856-4184	-7.798	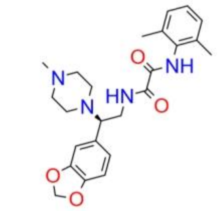	438.53	2.75	0
K292-1641	-7.534	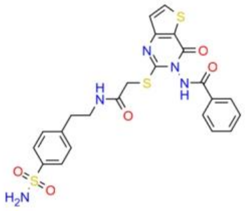	543.63	2.47	0
K784-3476	-7.517	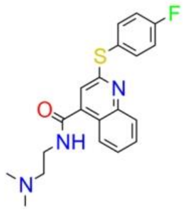	369.46	4.23	0
D337-1446	-7.511	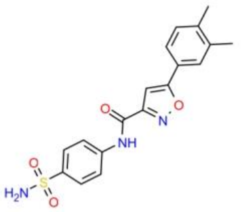	371.41	2.98	0
V006-6582	-7.132	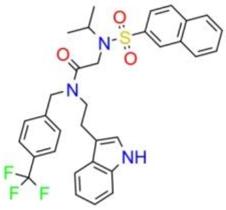	607.69	7.06	0
V014-3026	-7.254	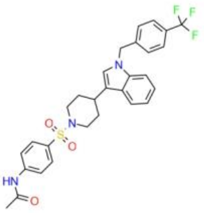	555.62	6.51	0
Y300-1847	-7.52	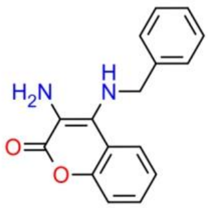	363.45	1.13	0

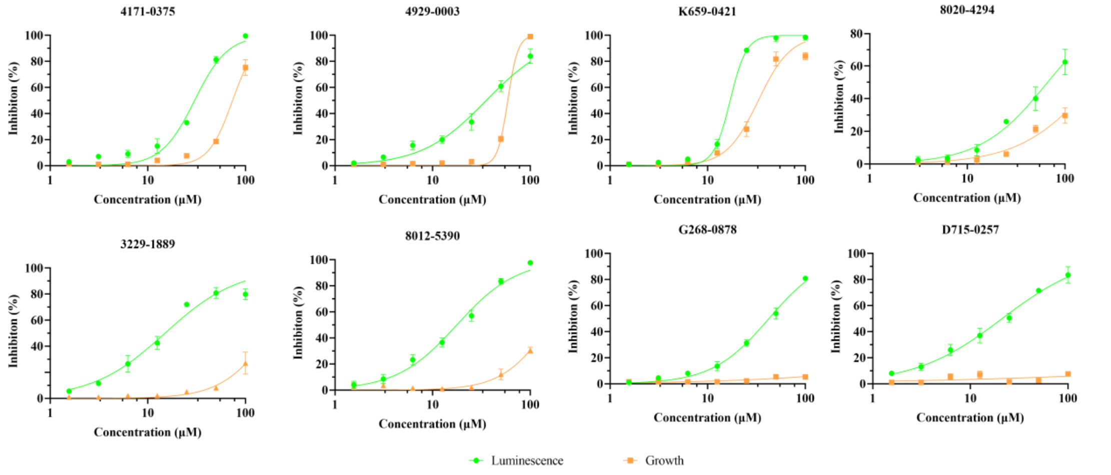

Figure S1. Dose–response curves of the eight positive hit compounds in an AI-2 QS interference assay with WHQ02 (*Escherichia coli* BL21 ΔTolC pWHQ01). Data points represent the mean ± SD from three independent experiments (*n* = 3).

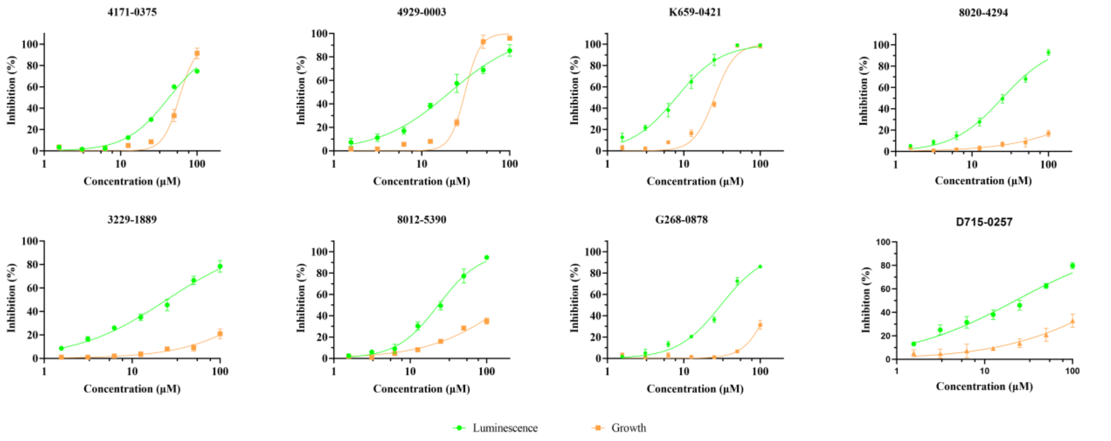

Figure S2: Dose–response curves of the eight positive hit compounds in an AI-2 QS interference assay with WHQ01 (*Salmonella typhimurium* ATCC 202165 ΔTolC pWHQ01). Data points represent the mean ± SD from three independent experiments (*n* = 3).
